# Towards a Reconceptualization of Striatal Interactions Between Glutamatergic and Dopaminergic Neurotransmission and Their Contribution to the Production of Movements

**DOI:** 10.2174/157015909788848893

**Published:** 2009-06

**Authors:** Hélène N David

**Affiliations:** NNOXe Pharmaceuticals, 3107 Avenue des Hôtels, Suite 18C, Québec, QC, G1W 4W5, Canada

**Keywords:** Dopamine-glutamate striatal interactions, locomotor activity, goal-oriented movements, nucleus accumbens, striatum.

## Abstract

According to the current model of the basal ganglia organization, simultaneous activation of the striato-nigral direct pathway by glutamatergic and dopaminergic neurotransmission should lead to a synergistic facilitatory action on locomotor activity, while in contrast activation of the indirect pathway by these two neurotransmittions should lead to antagonistic effects on locomotor activity. Based on published data, as a break with the current thinking, we propose a reconceptualization of functional interactions between dopaminergic and glutamatergic neurotransmission. In this model, dopaminergic neurotransmission is seen as a motor pacemaker responsible for the basal and primary activation of striatal output neurons and glutamate as a driver providing a multiple combination of tonic, phasic, facilitatory and inhibitory influxes resulting from the processing of environmental, emotional and mnesic stimuli. Thus, in the model, glutamate-coded inputs would allow tuning the intrinsic motor-activating properties of dopamine to adjust the production of locomotor activity into goal-oriented movements.

## 1. FUNCTIONAL NEUROANATOMY BETWEEN GLUTAMATERGIC AND DOPAMINERGIC NEUROTRANSMISSION IN THE STRIATUM COMPLEX (FIGS. 1 & 2)

The striatum including its dorsal and ventral parts, respectively the caudate-putamen and the nucleus accumbens, is the major input structure of the basal ganglia, which are a set of subcortical structures that is now widely accepted to contribute not only to the control of motor activity and movements but also to the processing of cognitive and ‘limbic’ (emotional and motivational) functions. While the motor system directly mediates the production of movements, the limbic system is involved in the elaboration of emotions and motivations, which basic drives can influence the production of movements and therefore the pertinence and efficacy of actions and goal-oriented behaviors [39,42]. Within the framework of interactions between the limbic system and the motor system, the nucleus accumbens, which corresponds to the limbic striatum, is considered to be a critical interface between the motor system and the limbic system in the brain. While the shell of the nucleus accumbens is generally thought to be associated more particularly with visceral responses, the core of the nucleus accumbens, which possesses similarities with the dorsal striatum (i.e. the caudate-putamen) both in terms of motor functions and connectivity, is believed to be allied more with motor functions [8,42].

From an anatomofunctional point of view, the striatum complex receives neuronal projections from all areas of the cerebral cortex and from limbic and other brain structures. These inputs originating from the cerebral cortex, the centromedian and parafascicularis nucleus of the thalamus, the hippocampus, and the amygdala are excitatory and use glutamate as a neurotransmitter. These inputs converge to the limbic and motor striatum where they make synaptic contacts at pre- and postsynaptic levels with striatal spiny output neurons [29,32,69,70] and striatal aspiny GABAergic and cholinergic interneurons [47,52]. In addition to these excitatory inputs, further inputs from outside and from within the striatum provide additional neuronal influxes, the function of which is thought to modify the responsiveness of striatal projection neurons to glutamateric inputs. Among the influxes originating from outside the striatum are dopaminergic inputs from the substantia nigra pars compacta, the ventral tegmental area, and the retrorubral nucleus [31,61], as well as serotoninergic inputs from the raphe nuclei [79]. Further evidence supporting such a dual regulation of striatal projection neurons is that glutamatergic and dopaminergic terminals directly converge on the dendritic spines of the same striatal projection neurons [13,69]. Like striatal output neurons, striatal interneurons are quite evenly distributed within the striatum [41]. Most of them make synapses with striatal projection neurons and thus may be viewed as intermediate relays between neuronal inputs from outside the striatum and striatal projection neurons. In turn, the striatum complex projects back upon the frontal lobe of the cortex *via* direct and indirect topographically organized pathways those which pass through the thalamus to organize goal-oriented movements [3-7,24,67]. Both the direct and indirect pathways utilize the same neuronal cell type, namely the striatal spiny projection neuron that represents approximately 95 % of the neuronal population of the striatum [10,26,44,85]. Spiny output neurons are quite evenly distributed within the striatum complex and are both the major input targets, although not exclusively, and the major output neurons of the striatum. Their connections are thus the major determinant of the functional organization of the striatum. Striatal projection neurons all use the inhibitory neurotransmitter γ-aminobutyric acid (GABA). Striatal output neurons of the “direct pathway” project directly from the striatum to the substantia nigra pars reticulata. In contrast, striatal output neurons of the “indirect pathway” connect the striatum to the substantia nigra pars reticulata *via* intermediate synaptic connections in the globus pallidus and the subthalamic nucleus. Projections from the striatum to the globus pallidus and from the globus pallidus to the subthalamic nucleus are GABAergic. Neurons originating from the subthalamic nucleus are glutamatergic, and project to the substantia nigra pars reticulata. Finally, the substantia nigra pars reticulata, the major outputput structure of the basal ganglia in rodents, sends GABAergic fibers to the thalamus, which in turn projects back to the motor cortex and other cortical area of the frontal lobe through excitatory glutamatergic fibers.

Despite the dual regulation of striatal output neurons by dopaminergic and glutamatergic inputs, there are very few axo-axonic synapses between glutamatergic and dopaminergic terminals throughout the entire striatum complex [13,31, 66,69,77]. Although this lack of axo-axonic synapses between glutamatergic and dopaminergic terminals limits the possibility of presynaptic interactions in a classical manner, dopamine and glutamate spilled over from the synaptic cleft modulate each other’s release through diffusion of neurotransmitters away from the synapse, leading to subsequent activation of extrasynaptic heteroreceptors through the socalled volume neurotransmission [69,77], and further interact by modulating in concert the activity of striatal output neurons.

## 2. STRIATAL MOTOR INTERACTIONS BETWEEN GLUTAMATERGIC AND DOPAMINERGIC NEUROTRANSMISSION: CURRENT THINKING

The current thinking on striatal motor interactions between glutamatergic and dopaminergic neurotransmission has come mainly from the work of Albin *et al.* [3] and DeLong [25] who first described a scheme, known as the model of basal ganglia, which was originally proposed to allow structuring glutamatergic and dopaminergic interactions in the dorsal striatum, i.e. the caudate and the putamen, as regards to the production of locomotor activity and movements. Due to its considerable success, this model has been adopted as a general principle and mode of reasoning and thinking as regards to glutamatergic and dopaminergic striatal motor interactions.

**Fig. (3).**
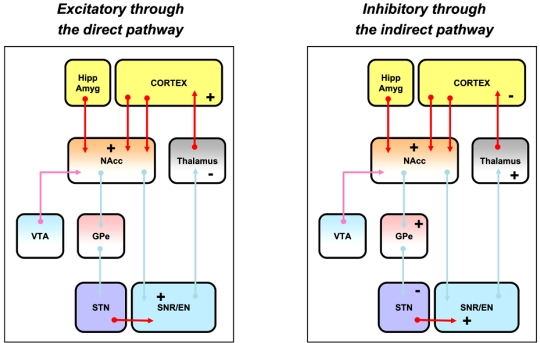
Current Model of Basal Ganglia - Glutamatatergic Control.

According to the current thinking as regards to striatal motor function, activation by glutamate (Fig. **3**) of the striatal GABAergic output neurons that project directly to the substantia nigra pars reticulata (the striato-nigral output neurons that form the direct pathway) is thought to provide a direct inhibitory action on the substantia nigra pars reticulata GABAergic neurons, the inhibition of which in turn leads to a disinhibition of the glutamatergic neurons involved in motor activities that project from the thalamus to the cortex; behaviorally, this allows the production of locomotor activity and movements. On the contrary, activation of the striatopallidal GABAergic output neurons, which project indirectly to the substantia nigra pars reticulata through a trisynaptic link and form the so-called indirect pathway, is believed to lead to an inhibition of the GABAergic neurons of the globus pallidus, thereby leading to a disinhibition of the subthalamic nucleus glutamatergic neurons and as a consequence to an activation of the subtantia nigra pars reticulata GABAergic neurons that project to the thalamus; overall, this provides an inhibitory effect on thalamo-cortical glutamatergic neurons involved in motor activities and thereby reduces the production of locomotor activity and movements. Although the output activity of the basal ganglia is influenced by the opposing effects of glutamatergic neurotransmission at the direct and indirect pathways, it is assumed that the direct pathway exerts a predominant control on locomotor activity.

Support for this model has been obtained from experiments in non-human primates that have demonstrated that the activation of striato-nigral and striato-pallidal output neurons, respectively, facilitate and suppress the production of motor activity and movements [4,5,25]. In addition, in rodents, further support for the predominant role of striatonigral output neurons in the control of motor activity has been obtained from pharmacological studies that have consistently shown that an intrastriatal injection of glutamate or glutamate receptor agonists in the caudate-putamen or the nucleus accumbens induces an increase in locomotor activity [11,12,14,15,20,22,23,27,34,36,48,64,68,71,72,75,82,86 but 45, 49,65]. However, leading to the conclusion that the role in the control of motor activity of glutamate inputs within the striatum is probably much more complex than that proposed by the model above, other studies have shown that the intrastriatal injection of glutamate receptor antagonists in the caudate-putamen or the nucleus accumbens also results in an increase in locomotor activity [12,15,20,23,27,36,43,50,59, 65,68,75,86 but 18].

**Fig. (4).**
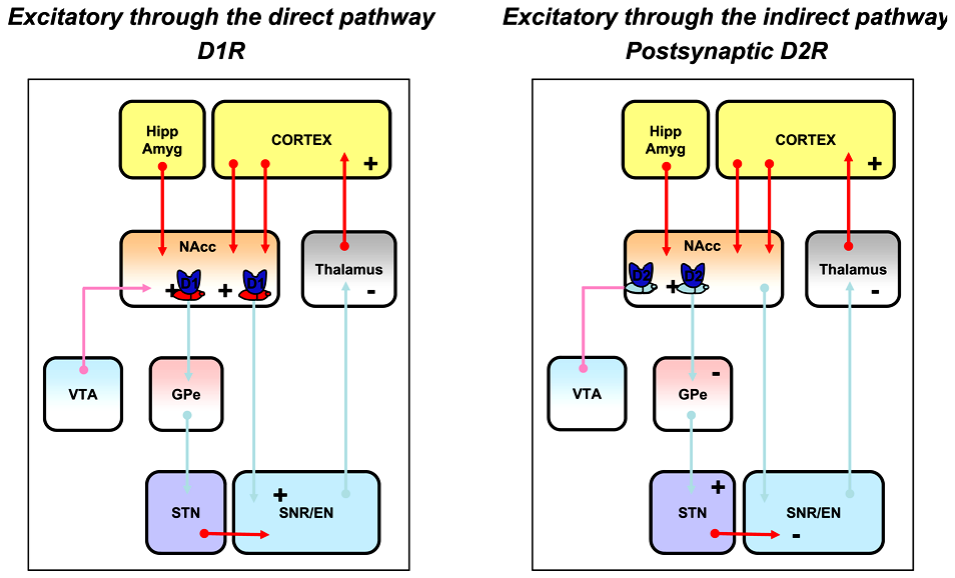
Current Model of Basal Ganglia - Dopaminergic Control.

The model of basal ganglia organization and motor function also proposes that dopamine modulates glutamatergic inputs by exerting a dual effect on striatal GABAergic output neurons (Fig. **4**): exciting striatal output neurons of the direct pathway, which mainly express D1-like receptors, and inhibiting striatal output neurons of the indirect pathway, which mainly express D2-like receptors. The segregation of D1-like- and of D2-like postsynaptic receptors has provided the anatomical basis for the D1-like receptor-mediated excitatory effect of dopamine on striato-nigral neurons that form the direct pathway and the D2-like receptor-mediated inhibitory action of dopamine on striato-pallidal neurons that constitute the first synaptic link of the indirect pathway. According to the anatomical organization of the basal ganglia from the striatum complex to the motor cortex, the model predicts that activation of either D1-like receptors or D2-like postsynaptic receptors leads to the production of locomotor activity as follows: activation by dopamine of the striato-nigral GABAergic output neurons is thought to provide a direct inhibitory action on the substantia nigra pars reticulata GABAergic neurons. This inhibition in turn leads to a disinhibition of the glutamatergic neurons involved in motor activities that project from the thalamus to the cortex and thereby to the production of locomotor activity and movements. Alternatively, activation of the striato-pallidal GABAergic output neurons is believed to result in a dishinibition of the globus pallidus GABAergic neurons, leading to a subsequent inhibition of the subthalamic nucleus glutamatergic neurons and to a reduction in activity of the subtantia nigra pars reticulata GABAergic neurons that project to the thalamus. This inhibition in turn finally leads to a disinhibition of the glutamatergic neurons involved in motor activities that project from the thalamus to the cortex and thereby to the production of locomotor activity and movements.

A large body of evidence supports a functional synergy between D1-like and D2-like postsynaptic receptors that are mainly located on striato-nigral- and striato-pallidal output neurons, respectively [1,2,46,80,81,84]. At other times, activation of each dopaminergic receptor subtype produces antagonistic effects [57]. Accordingly, the very vast majority of behavioral motor studies has shown: (i) activation by specific agonists of D1-like receptors results in an increase in locomotor activity [18,20-23,28,33,53,54,73,87 but 55,60]; (ii) co-activation of D1-like- and D2-like receptors – a pharmacological condition shown to be required for the full expression of the postsynaptic effects of D2-like receptor agonists because of the enabling role of D1-like receptors on D2-like postsynaptic receptor activation – results in synergistic motor effects leading to a locomotor activity much more greater than that produced by the activation of D1-like receptors alone, thought to be mainly mediated by D2-like postsynaptic receptors [16,18,19,23,28,30,33,37,40,60] and reported as such in the text below; (iii) activation in the caudate-putamen or the nucleus accumbens of D2-like receptors alone – a pharmacological condition thought to reflect mainly D2-like presynaptic receptor mechanisms [38,76,83] results at a very few exceptions [24] in a decrease in locomotor activity [16,18,20-24,33,55,63,71,73 but 51 ,60]. In addition, support for the model of basal ganglia has been obtained from studies that have consistently shown that blockade of dopaminergic receptors by intrastriatal infusion of D1-like and/or D2-like receptor antagonists resulted in a decrease in locomotor activity [35,53,58,60,78].

According to this model, simultaneous activation of glutamatergic receptors and D1-like receptors within the striatum complex should lead to a synergistic facilitatory action on locomotor activity through the direct striato-nigral pathway, while activation of glutamatergic receptors and D2-like receptors should lead to antagonistic effects on locomotor activity through the indirect pathway. However, it is clear from the data summarized in Table **1**, obtained from *focal* injection studies performed in the caudate-putamen or the nucleus accumbens of “intact” animals, that an attempt to compel behavioral motor interactions between glutamatergic and dopaminergic neurotransmission into the current model of basal ganglia organization, and an exclusively excitatory role of glutamatergic neurotransmission, would fail to provide a reliable framework of functional interactions between both neurotransmitter systems in the striatum complex.

## 3. TOWARDS A RECONCEPTUALIZATION OF FUNCTIONAL STRIATAL INTERACTIONS BETWEEN GLUTAMATERGIC AND DOPAMINERGIC NEUROTRANSMISSION AND THEIR ROLE IN THE PRODUCTION OF MOVEMENT

In this section, we will develop particular points that we believe to be important to layout by which glutamatergic and dopaminergic neurotransmissions may interact within the striatum complex to modulate striatal projection neurons and thereby the production of locomotor activity and movements in “intact” animals. Our analysis is based on an overview of previous published papers that have investigated in non-lesioned animals functional interactions between glutamatergic and dopaminergic neurotransmission in the caudate-putamen and the core of the nucleus accumbens using focal (intrastriatal) injection of glutamate and dopamine receptor ligands. Behavioral systemic injection studies with glutamatergic and dopaminergic neurotransmission were omitted intentionally because this technique is not without drawbacks since the pharmacological effects of systemic injection are diffuse, thereby altering many other brain structures that are involved in motor control throughout the brain.

Modulation by glutamatergic neurotransmission of locomotor responses produced by activation of dopaminergic postsynaptic receptors is in fact very heterogeneous and contrasts with the current model of basal ganglia striatal motor function, which assumes that glutamate exerts a facilitatory and an inhibitory action on locomotor activity through striato-nigral and striato-pallidal projection neurons respectively. Based on the behavioral motor findings obtained from focal injection studies that are summarized in Table **1** and from other electrophysiological investigations [17], a more reliable and productive approach may be to fit the effects of glutamatergic neurotransmission into a modulatory, either facilitatory or inhibitory, role in a way that depends on the type of receptor involved. This proposal clearly breaks away from the current model of striatal motor function and the current theory about the role and function of glutamate, which is viewed as an excitatory neurotransmitter that produces activation of striato-nigral and striato-pallidal projection neurons. Though such a modulatory function of glutamatergic neurotransmission within the striatum complex may lead to heated debates and conflicts of opinion, there is now evidence that, depending on the type of receptor involved, activation of glutamatergic receptors can produce either a facilitatory or an inhibitory action (or have no action) on the locomotor responses produced by activation of dopaminergic postsynaptic receptors. Reciprocally, there is now a growing body of evidence that not only dopaminergic neurotransmission modulates glutamate release in the striatum, but that glutamatergic neurotransmission, depending on the type of receptor involved, also exerts a modulatory, either facilitatory or inhibitory, action on striatal dopamine release in *ex vivo* and *in vivo* models [24].

**Fig. (5).**
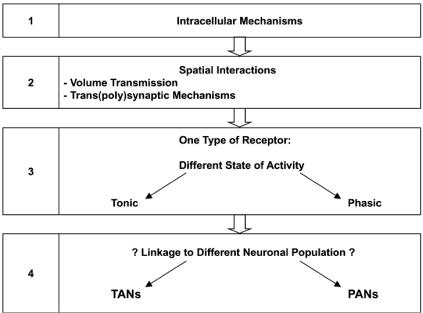
What can emerge from functional studies?

If glutamate is considered as a neurotransmitter which activation can positively and negatively control both striatonigral and striato-pallidal output neurons, and *in fine* facilitate or inhibit the production of locomotor activity and movements mediated by D1-like receptor- or D2-like postsynaptic receptor activation, it could be predicted that the types of receptors and glutamatergic pathway (e.g. from the prefrontal cortex, the hippocampus or the amygdala) activated would determine the direction in which functional interactions between glutamatergic and dopaminergic neurotransmission occur. However, the mechanisms by which couplings of specific glutamatergic and dopaminergic receptor subtypes occur cannot be foreseen with enough accuracy at this time, and only hypotheses can be drawn. Coupling possibilities may involve intracellular interactions on common neurons as well as spatial interactions through trans (poly)synaptic mechanisms. For example, co-activation of NMDA and D1-like receptors produces behavioral and electrophysiological synergistic effects while co-activation of NMDA and D2-like receptors produces antagonistic effects. Consistent with intracellular mechanisms, it is well known that NMDA receptors and D1-like receptors tap into the same transduction system that is the adenosine monophosphate cyclic – phosphokinase A transduction system, while D2-like receptors inhibit it. Likewise, also consistent with intracellular mechanisms, activation of group III mGlu receptors inhibits the adenosine monophosphate cyclic – phosphokinase A transduction system and behaviorally opposes and favors the locomotor-activating properties of D1-like receptor activation and D2-like postsynaptic receptor activation, respectively. Another possibility is spatial interactions including volume neurotransmission and trans(poly)synaptic mechanisms, in which glutamatergic and dopaminergic receptors interact with each other distally. This possibility (Fig. **5**) should be considered with particular interest when functional interactions between glutamatergic and dopaminergic neurotransmission do not fit with compatible intracellular mechanisms, even if they do fit with the current model of striatal motor function. For example, co-activation of D1-like receptors and group II mGlu receptors produces behavioral motor synergistic effects as predicted by the current model of basal ganglia motor function, but are respectively positively and negatively linked to adenylate cyclase. Therefore, intracellular interactions between these receptors on common neurons do not appear tenable and spatial interactions through volume neurotransmission mechanisms should have to be considered. Consistent with such mechanisms, ultrastructural studies have shown that group II, in contrast with group I and group III, mGlu receptors have no close apposition with synapses [74]. Likewise, co-activation of non-NMDA and D1-like receptors produces motor antagonistic effects while co-activation of non-NMDA and D2-like receptors produces synergistic effects. These effects do not fit with the current model of striatal motor function and the fact that non-NMDA, like NMDA, receptors produce postsynaptic excitatory currents at striatal output neurons [56]; therefore, the possibility that glutamatergic and dopaminergic neurotransmission interact spatially through trans (poly)synaptic mechanisms to modulate striatal projection neurons must be considered. The same principles of data analysis and interpretation are also applicable to blockade of glutamatergic receptors. In that way, the importance of investigating the effects of receptor blockade clearly has to be emphasized. Indeed, it is obvious from the data summarized in Table **1** that the effects produced by the activation or blockade of glutamatergic receptors on D1-like receptor- and D2-like pre- and postsynaptic receptor-mediated locomotor responses are not always reciprocal. This may be due to the fact that, for a given subtype of receptors, certain receptors are tonically activated while others are “silent” and can be phasically activated. Indeed, if a receptor is tonically actived, it cannot be further activated by a receptor agonist but can be blocked by a receptor antagonist; likewise, if a receptor is silent, it cannot be further blocked by a receptor antagonist but can be activated phasically by a receptor agonist. We propose that this may provide informations about the neuroanatomical localization within the striatum complex of the glutamatergic receptors studied, either on phasically activated neurons (PANs), such as the spiny output neurons that exhibit increases in firing in relation to movement, or on tonically activated neurons (TANs) such as the cholinergic interneurons that spike in a tonic and irregular fashion [9].

Because all glutamatergic and dopaminergic receptor subtypes are expressed in striatal projection neurons, we believe that priority of interpretation must be given to intracellular mechanisms. However, spatial mechanisms must also be considered whether or not intracellular mechanisms explain functional interactions between glutamatergic and dopaminergic neurotransmission. Doubtlessly, such a complex combination of modulatory properties can no longer be regarded as a bulk system where glutamatergic inputs are all considered as excitatory. As a basis for an extended, but not yet complete, model of glutamatergic innervation of striatal output neurons, we propose that depending on their anatomical origin glutamatergic terminals may tap onto specific, and not all, glutamatergic receptor subtypes. Support for this are recent studies [62] that have shown that a reversible inhibition by the local anesthetic lidocaine of the prefrontal cortex, the hippocampus complex or the basolateral amygdala (which are brain structures that project glutamatergic fibers toward the striatum complex) has differential facilitatory and inhibitory effects on hyperlocomotion induced by activation in the nucleus accumbens of D1-like receptors or D2-like postsynaptic receptors that mimick those of glutamatergic receptor activation or blockade.

**Fig. (6).**
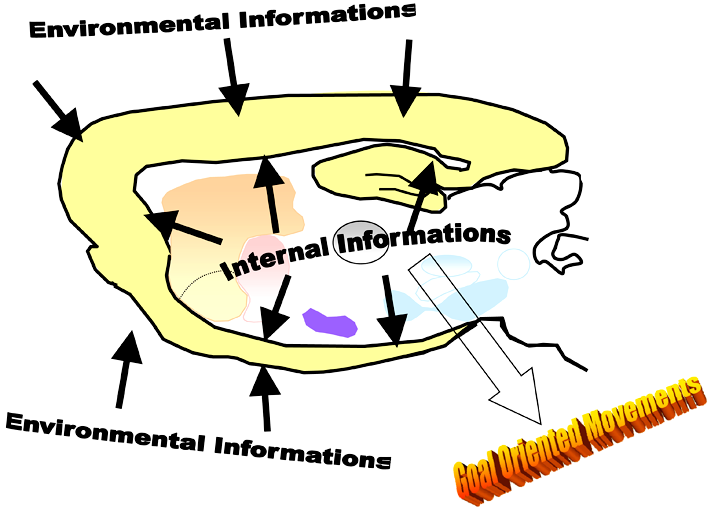
Glutamate-coded inputs would allow tuning the intrinsic motor-activating properties of dopamine to adjust the production of locomotor activity into goal-oriented movements.

In addition, according to the current view as regards to dopamine-glutamate interactions in the striatum complex, glutamate is often seen as an excitatory neurotransmitter that is modulated by dopamine. However, in addition of the experimental evidence summarized in Table **1**, there are neuroanatomical arguments to suggest that glutamate may be viewed on the contrary as a modulator, or better a *driver*, of dopaminergic neurotransmission (Fig. **6**). Glutamatergic pathways that project to the striatum complex mainly originate from the whole cortex and limbic structures, such as the amygdala and the dorsal and ventral hippocampus. The cortex and limbic structures are well known to play a key role in the processing of extrinsic (environmental) and intrinsic (emotional and mnesic) stimuli. Dopaminergic pathways that project to the dorsal and ventral striatum mainly originate from the substantia nigra pars compacta, the ventral tegmental area and retrorubral nucleus, which deeper brain structures are thought to be only slighly involved in the processing of extrinsic and intrinsic stimuli compared to the cortex and limbic structures. Therefore, in contrast with the current thinking of a dopaminergic modulation of glutamatergic neurotransmission, we propose that dopaminergic neurotransmission should be seen as a subcortical-infralimbic motor pacemaker responsible for the basal and primary activation of striatal output neurons, and glutamate as a driver providing facilitatory and inhibitory inputs resulting from the processing of extrinsic and intrinsic stimuli by the cortical areas and limbic structures. These glutamate-mediated facilitatory and inhibitory inputs would allow modulating the motor-activating properties of dopamine at striatal output neurons in order to transform and adjust the production of motor activity into goal-oriented movements. As a result, the relevance and accuracy of goal-oriented movements depend on the appropriate processing of extrinsic (environmental) and intrinsic (emotional and mnesic) stimuli; and this is the reason why, for instance, strategic processes, perceptive awareness, and learning influence goal-oriented movements. However, because infusion in the striatum complex of either glutamate receptor agonists or antagonists consistently results in an increase of locomotor activity (see above), thereby indicating phasic and tonic facilitatory properties of glutamatergic neurotransmission *per se*, it is likely that the direction of the glutamatergic modulation of dopaminergic neurotransmission would depend on the level of the dopaminergic tone within the striatum complex. In other words, glutamatergic inputs would adapt their inputs as a function of outer and inner factors to the body to modulate dopaminergic neurotransmission and to produce accurate goal-oriented movements.

## CONCLUSION

The principal function of oncoming glutamatergic and dopaminergic fibers that innerve the striatum complex is to modulate the activity and responsiveness of striatal output neurons. The present article emphasizes from a behavioral motor point of view the complexity of interactions between glutamatergic and dopaminergic neurotransmission, and further propose a reconceptualization of these interactions in order to induce debate. Clearly, in the future, a great amount of research will be necessary to better define and understand intrastriatal interactions between these neurotransmitter systems. However, we believe that there is enough theroretical and empirical evidence to propose an extended, receptor and pathway-based, model of basal ganglia motor function, in which striatal dopaminergic neurotransmission can be seen as a motor pacemaker responsible for the basal and primary activation of striatal output neurons, and glutamate as a driver providing a multiple combination of tonic, phasic, facilitatory and inhibitory influxes resulting from the processing of extrinsic environmental and intrinsic emotional and mnesic stimuli. In this framework, and in contrast with the current thinking as regards to glutamate function, glutamate-coded inputs would allow adjusting the intrinsic motor-activating properties of dopamine in order to fine-tune the production of motor activity into goal-oriented movements.

## Figures and Tables

**Fig. (1). Basal Ganglia - Dorsal Pathways.**
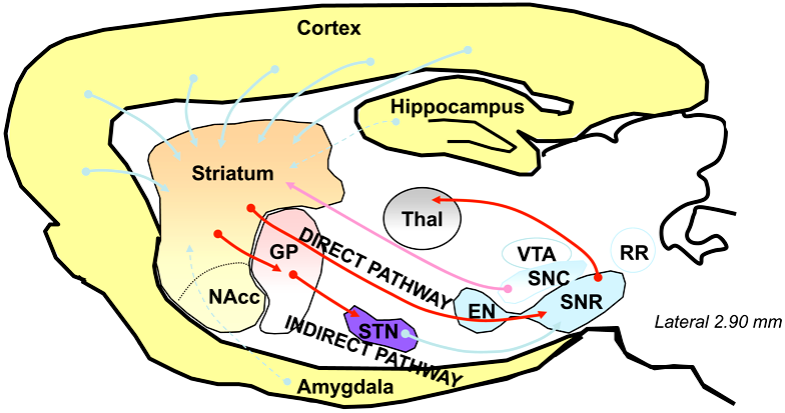
The dorsal striatum receives cortical informations and transfer them to the output structures of the basal ganglia: the entopedoncular nucleus (EN) and the subtantia nigra *pars reticulata* (SNR) through the direct and indirect pathway. Then, the information is sent to the frontal cortex through the thalamus. STN: subthalamic nucleus; NAcc: Nucleus Accumbens; SNC: subtantia nigra pars compacta; Thal: Thalamus; RR: retrorubral nucleus; GP: Globus Pallidus; VTA: ventral tegmental area. voie glutamatergique ; voie GABAergique; voie dopaminergique.

**Fig. (2). Basal Ganglia - Ventral Pathways.**
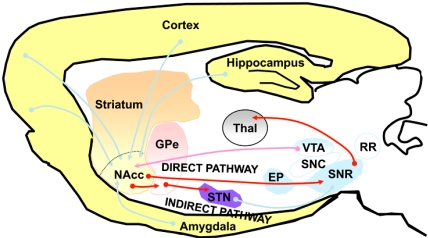
The nucleus accumbens receives cortical informations and transfer them to the output structures of the basal ganglia: the entopedoncular nucleus (EN) and the subtantia nigra *pars reticulata* (SNR) through the direct and indirect pathway. Then, the information is sent to the frontal cortex through the thalamus. STN: subthalamic nucleus; NAcc: Nucleus Accumbens; SNC: subtantia nigra pars compacta; Thal: Thalamus; RR: retrorubral nucleus; GP: Globus Pallidus; VTA: ventral tegmental area. voie glutamatergique ; voie GABAergique; voie dopaminergique.

**Table 1.** The Nucleus Accumbens: When Modelization Meets Experimental Research

Effect of Glutamate Receptor Ligands on Locomotor Activity Produced by Dopamine Receptor Activation	As Predicted by the bg Model	Experimental Results	References
NMDA receptor activation on D1-like receptor activation NMDA receptor blockade on D1-like receptor activation	+ –	+ n.e. –	[23] [86] [23]
NMDA receptor activation on D2-like receptor activation NMDA receptor blockade on D2-like receptor activation	– +	– –	[23] [23]
Non-NMDA receptor activation on D1-like receptor activation Non-NMDA receptor blockade on D1-like receptor activation	+ –	– – n.e.	[23] [23] [18]
Non-NMDA receptor activation on D2-like receptor activation Non-NMDA receptor blockade on D2-like receptor activation	– +	+ –	[23] [23;40]
Group I mGlu receptor activation on D1-like receptor activation Group I mGlu receptor blockade on D1-like receptor activation	+ –	+ –	[63] [20]
Group I mGlu receptor activation on D2-like receptor activation Group I mGlu receptor blockade on D2-like receptor activation	– +	– n.e	[63] [20]
Group II mGlu receptor activation on D1-like receptor activation Group II mGlu receptor blockade on D1-like receptor activation	+ –	+ n.e.	[21] [21]
Group II mGlu receptor activation on D2-like receptor activation Group II mGlu receptor blockade on D2-like receptor activation	– +	n.e. –	[21] [21]
Group III mGlu receptor activation on D1-like receptor activation Group III mGlu receptor blockade on D1-like receptor activation	+ –	– +	[22] [63]
Group III mGlu receptor activation on D2-like receptor activation Group III mGlu receptor blockade on D2-like receptor activation	– +	+ +	[22] [63]
